# Do Budget Cigarettes Emit More Particles? An Aerosol Spectrometric Comparison of Particulate Matter Concentrations between Private-Label Cigarettes and More Expensive Brand-Name Cigarettes

**DOI:** 10.3390/ijerph19105920

**Published:** 2022-05-13

**Authors:** Greta Gerlach, Markus Braun, Janis Dröge, David A. Groneberg

**Affiliations:** Institute of Occupational Medicine, Social Medicine and Environmental Medicine, Goethe University Frankfurt, Theodor-Stern-Kai 7, D-60590 Frankfurt am Main, Germany; greta.gerlach@gmx.de (G.G.); droege@med.uni-frankfurt.de (J.D.); arbsozmed@uni-frankfurt.de (D.A.G.)

**Keywords:** environmental tobacco smoke, passive smoke, private brands, store brands, smoking behavior, indoor air

## Abstract

Private-label cigarettes are cigarettes that belong to the retailer itself. Private-label cigarettes from discounters or supermarkets are cheaper than brand-name cigarettes, and their lower price has allowed them to garner an ever-increasing share of the tobacco product market, especially among lower socioeconomic groups. Particulate matter (PM), a considerable component of air pollution, is a substantial health-damaging factor. Smoking is the primary source of PM in smokers’ homes. In a 2.88 m^3^ measuring chamber, the PM emission fractions PM_10_, PM_2.5_, and PM_1_ from three private-label cigarette brands and three brand-name cigarette brands with identical nicotine, tar, and carbon monoxide content were measured and compared to those of a reference cigarette by laser aerosol spectroscopy. All cigarette brands emitted PM in health-threatening quantities. The measurement results ranged from 1394 µg/m^3^ to 1686 µg/m^3^ PM_10_, 1392 µg/m^3^ to 1682 µg/m^3^ PM_2.5_, and 1355 µg/m^3^ to 1634 µg/m^3^ PM_1_, respectively. Only one private-label brand differed significantly (*p* < 0.001) from the other cigarette brands, which were tested with slightly lower PM levels. All other brands differed only marginally (not significant, *p* > 0.05) from one another. Significant (*p* < 0.05) negative correlations between private-label and brand-name cigarettes were found for PM_10_, PM_2.5_, and PM_1_ when accounting for tobacco filling densities, and for PM_1_ when accounting for filter lengths. The especially health-hazardous fraction PM_1_ accounted for the largest proportion of PM emissions from the cigarettes tested. The results of this study suggest that- cheaper tobacco products are as harmful as more expensive ones, at least regarding PM emissions. This highlights the importance of anti-smoking campaigns, especially for lower socioeconomic groups, where smoking is more widespread. Governments should reduce the price gap between cheap and more expensive tobacco products by implementing specific tobacco taxes. In such a case, at increasing prices of tobacco products, a downward shift to private-label cigarettes would probably decrease.

## 1. Introduction

More than 8 million people worldwide die each year from tobacco consumption. Of these deaths, about 1.2 million are associated with second-hand smoke (SHS) [1]. Tobacco smoke contains, among others, 250 toxic and 90 carcinogenic or potentially carcinogenic substances [2]. It is well-known that smokers have a strikingly increased risk of developing diseases such as lung cancer, chronic obstructive pulmonary disease (COPD), or cardiovascular diseases [2]. The same is true for SHS exposure [2].

The level of particulate matter (PM) is a widely used marker for SHS exposure [3]. Smoking households have substantially increased PM pollution compared to non-smoking homes [4]. Since indoor spaces are usually less ventilated, PM load is frequently higher compared to outdoor air. PM consists of solid and liquid particles in the air. It is classified by the US Environmental Protection Agency (EPA) as PM_10_ (inhalable, ≤10 µm) and PM_2.5_ (fine inhalable, ≤2.5 µm) [5]. Accordingly, PM_1_ particles are smaller than 1 µm. PM is a primary factor that negatively affects air quality and is harmful to health. The World Health Organization (WHO) estimates that 8 million people die prematurely due to indoor and outdoor air pollution annually [6]. An increase in mortality and morbidity, especially due to respiratory and cardiovascular diseases, is associated with PM concentration and exposure duration [7]. The smaller the particles are, the deeper they can penetrate the respiratory tract, and the more dangerous they are to health [8].

Private-label brands, also known as private brands or store brands, are products that belong to the retailer itself. Private-label cigarettes from discounters or supermarkets are cheaper than brand-name cigarettes [9]. As a result of steadily increasing cigarette prices, cheaper, private-label cigarettes are likely to play an increasingly important role [9]. In Germany for example, the consumption of brand-name cigarettes decreased from 77.1 billion in 2008 to 63.3 billion in 2020, whereas the consumption of private-label cigarettes was at an approximate steady mean level of 9.43 billion cigarettes, with an increase in market share from 11.3% to 13.3% [10].

This paper compares the PM concentrations in SHS from cheaper private-label versions of specific cigarettes and the more expensive brand-name cigarettes with identical nicotine, tar, and carbon monoxide amounts. Can a relationship between measured PM values and cigarette prices be ascertained due to, e.g., potential differences in the quality of the components or the manufacturing processes?

## 2. Materials and Methods

### 2.1. Tobacco Products

In the test series, the PM concentrations in SHS of three different brand-name cigarette brands (Camel Yellow Filter, Marlboro Red, Nil Blue; each 0.34 EUR/cigarette) and three different private-label cigarette brands (Giants Red, Goldfield Red, Jakordia Red; 0.24 EUR/cigarette) were measured. All six tobacco products have the following ingredients in common (manufacturer’s data of nominal values; not validated): 0.8 mg nicotine, 10.0 mg tar, and 10.0 mg carbon monoxide (CO). The reference cigarette 3R4F from the University of Kentucky with 0.73 mg nicotine, 9.4 mg tar, and 12.0 mg CO per cigarette was taken as the standard of comparison [11]. Table 1 shows some characteristics of the cigarettes investigated. The database of tobacco additives from the Federal Ministry of Food and Agriculture of Germany provides additional information [12].

### 2.2. Measuring Chamber

The experiments took place in a 2.88 m^3^ glass-sealed measuring chamber. Two rubber gloves were embedded in a glass wall for lighting and extinguishing the cigarettes from outside the chamber by the investigator. An industrial radial fan was switched on for at least 5 min after each cigarette was smoked, to suck the smoke particles out of the chamber. During the smoking phases, the air vents were closed to minimize air exchange. The sensor Grimm model 1.154 measured the temperature and relative humidity (RH) during all PM measurements. The mean temperature during all PM measurements was 20.75 °C (SD 1.495), and the mean RH was 29.92% (SD 4.017).

### 2.3. Automatic Environmental Tobacco Smoke Emitter

The Automatic Environmental Tobacco Smoke Emitter (AETSE), a programmable smoke pump placed in the measuring chamber, was manufactured by Schimpf-Ing Trondheim (Norway) for the simulation of tobacco product smoking [13]. It consists of a 200 mL glass syringe, a microcontroller, and two valves, and connects via a polyamide tube to the mouthpiece of the cigarettes. A carriage moved by a stepper motor actuates the syringe, taking pulls on the tobacco product. The mainstream smoke is then pressed back into the chamber. The two valves control the airstream. In this way, puffs on the lit cigarettes are imitated. The smoldering cigarette produces side-stream smoke.

### 2.4. Laser Aerosol Spectrometer

The Laser Aerosol Spectrometer (LAS) model 1.109 from Grimm Aerosol Technik GmbH & Co. KG [14] continuously measured the quantity of airborne particles and the particle size distribution from the sucked-in sample air. The suction point was placed 38 cm beside the cigarette and the outlet valve was placed at the same height. SHS drawn into the LAS was previously diluted with compressed air at a ratio of 1:10, using the Grimm VKL mini dilution system (model 7.951) to protect the measuring cell of the LAS from excessive particle concentrations. The dilution ratio was taken into account during the data processing. Data could be displayed, among others, in dust mass fractions according to US EPA in PM_10_ and PM_2.5_ [5] plus PM_1_. The measurement spectrum ranged from 0.25 μm to 32 μm [14].

### 2.5. Smoking Protocol

The standardized smoking protocol is part of the Tobacco Smoke Particles and Indoor Air Quality (ToPIQ) studies [15,16,17,18,19,20,21,22]. It enables a reproducible and comparable measurement procedure of PM in SHS for different test series. It is a cycle consisting of three test phases: the combustion phase, the post-combustion phase, and the ventilation phase. The measurement data PM_1_, PM_2.5_, and PM_10_ were collected every six seconds. In the combustion phase, the cigarette was lit, followed by two initial puffs. Then, eight regular puffs followed with a frequency of two per minute. Each puff lasted three seconds and had a puff volume of 40 mL. After 4.5 min, the combustion phase was completed, and the cigarette was extinguished. For at least 5.5 min thereafter, the PM concentrations were measured in the post-combustion phase. After not less than 10 min after igniting the cigarette, the chamber was ventilated for at least 5 min to clean the air. This ensured exactly 10 min of data measurement and provided smoke-free air for the subsequent measurement.

**Figure 1:**
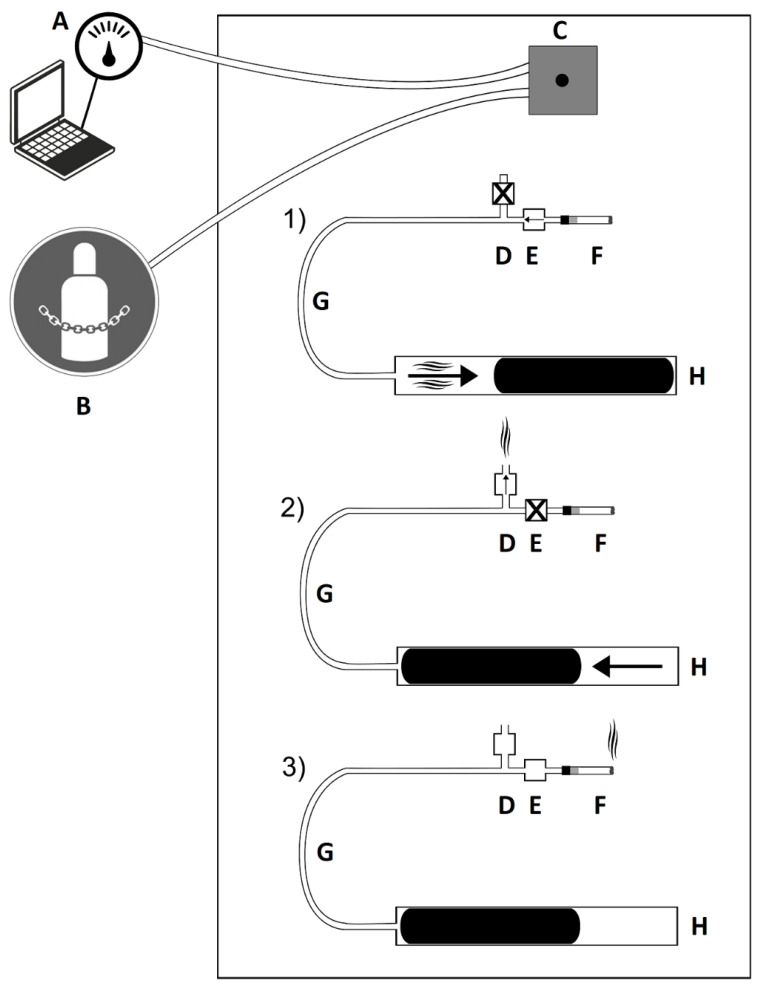
Diagram of the experiment. Outside the measuring chamber: A = Laser aerosol spectrometer (LAS) connected with computer. B = Compressed air supply. Inside the measuring chamber: C = Suction point and dilution system connected with LAS and compressed air supply via tubes. D = Outlet valve. E = Intake valve. F = Cigarette. G = Polyamide tube. H = Programmable smoke pump. (1) Smoke pump (H) takes a puff on the cigarette (F). Outlet valve (D) is closed. (2) Smoke pump (H) puffs out the smoke into the measuring chamber. The intake valve is closed. (3) Phase between two drags on the cigarette. Cigarette (F) smolders.

Figure 1 shows a diagram of the experiment with the three phases of tobacco product smoking.

### 2.6. Data Processing and Statistical Analysis

For statistical analysis of the 10 min data measurement of between 33 and 38 cigarettes each (Table 1), software GraphPad Prism version 9 (La Jolla, CA, USA) was used. The calculated mean concentrations (C_mean_) of PM_10_, PM_2.5_, and PM_1_ of all cigarettes investigated were tested for outliers (Grubb’s test) and normal distribution (passed). A one-way analysis of variance (one-way ANOVA) with Tukey’s multiple comparison test followed, testing the data of each cigarette against each other. The six private-label and respective brand-name cigarette brands were tested for correlation (Pearson) of the measured PM amounts with the ascertained filter lengths and tobacco weights and the calculated filling densities. The level of significance was set as *p* = 0.05.

## 3. Results

The measured PM C_mean_ values ranged from 1394 to 1686 µg/m^3^ (PM_10_), 1392 to 1682 µg/m^3^ (PM_2.5_), and 1355 to 1634 µg/m^3^ (PM_1_), respectively. The C_mean_ values of five cigarette brands investigated, including the reference cigarette 3R4F, differed only on a low level with no significance (*p* ≥ 0.5). Only the private-label brand Jakordia showed significantly lower C_mean_ values (*p* < 0.001) compared to all other brands tested. The three brand-name cigarettes investigated emitted significantly (*p* < 0.001) more PM than the Jakordia cigarette. Marlboro: +20.9% (PM_10_), +20.8% (PM_2.5_), and +20.6% (PM_1_); Nil: +19.6% (PM_10_), +19.4% (PM_2.5_), and +17.9% (PM_1_); Camel: +17.3% (PM_10_), +17.2% (PM_2.5_), and +18.2% (PM_1_). That was also true for the private-label brands Giants: +14.1% (PM_10_), +14.0% (PM_2.5_), and +13.8% (PM_1_), and Goldfield: +13.1% (PM_10_), +13.1% (PM_2.5_), and +12.1% (PM_1_). An overview of all C_mean_ values, percentage differences to the reference cigarette, and significance is shown in Table 2.

**Figure 2:**
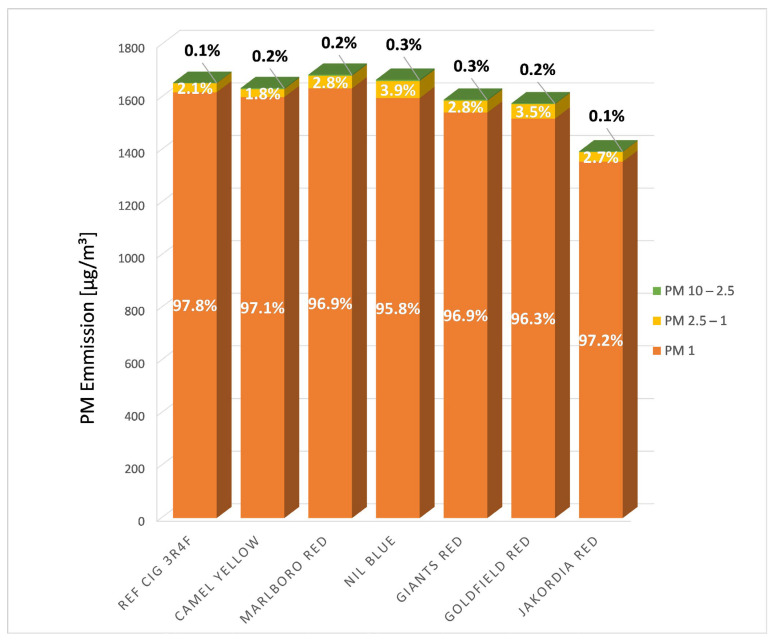
Distribution pattern of the mean concentrations (C_mean_) of all cigarette brands investigated. The percentages (rounded to one decimal place) of the particle fractions PM_10–2.5_, PM_2.5–1_, and PM_1_ are listed in the respective part of the column diagram.

All cigarette brands had in common that PM_1_ accounted for the largest share of PM, more than 95% (Figure 2).

**Figure 3:**
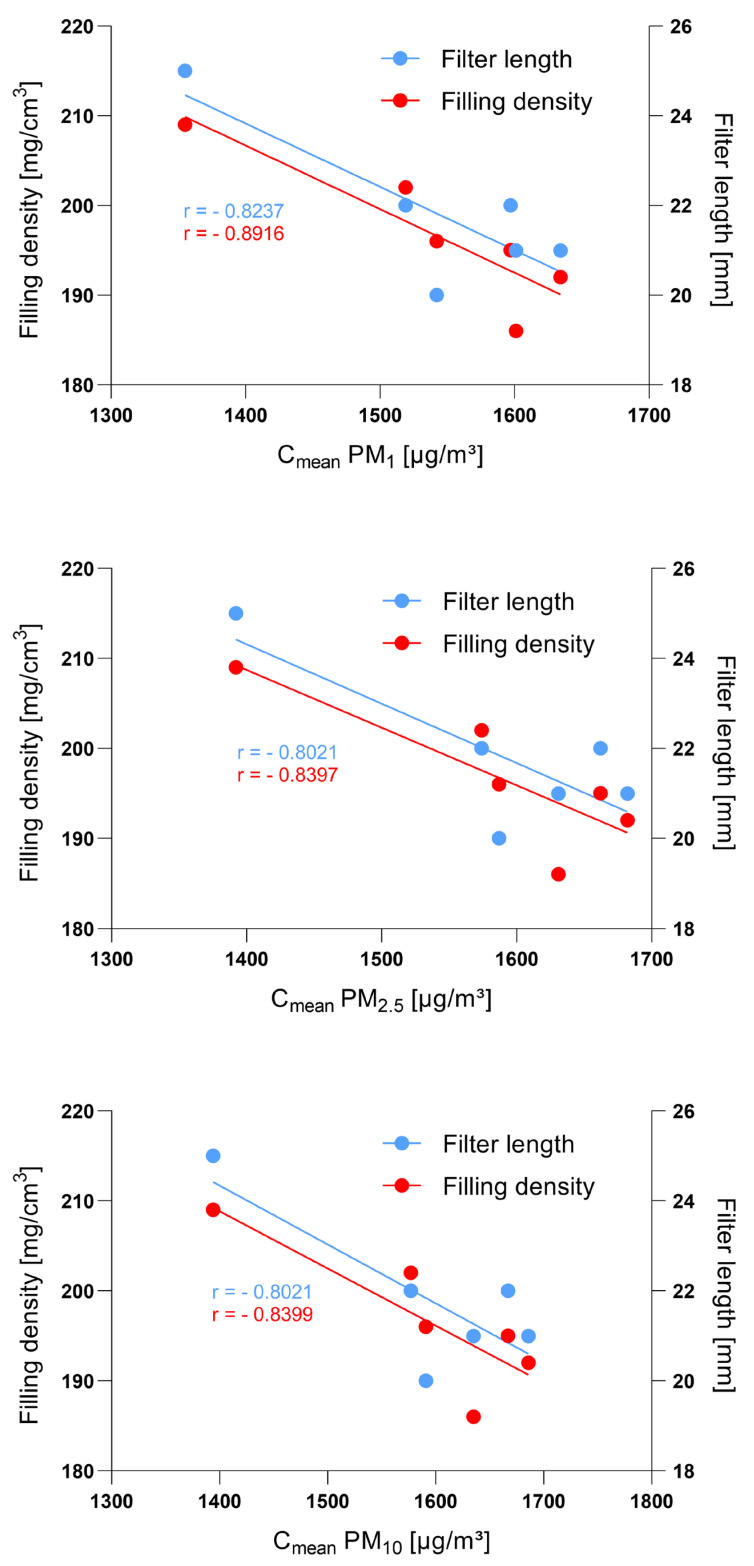
Correlations (Pearson) with linear regression lines of measured PM mean concentrations (C_mean_ PM_1_, PM_2.5_, and PM_10_) with ascertained filter lengths (blue, *p* (PM_1_) = 0.044, *p* (PM_2.5_ and PM_10_) > 0.05)) and calculated filling densities (red, *p* (PM_1_) = 0.017, *p* (PM_2.5_) = 0.037, *p* (PM_10_) = 0.036) of the private-label and brand-name cigarettes. *r* = Pearson’s correlation coefficient. *p* = Level of significance.

The measured PM values of the six private-label and brand-name cigarette brands each correlated significantly with the calculated filling densities (PM_10_: Pearson *r* = −0.8399, *p* = 0.036; PM_2.5_: Pearson *r* = −0.8397, *p* = 0.037; PM_1_: Pearson *r* = −0.8916, *p* = 0.017), but not with the ascertained tobacco weights (*p* > 0.05). PM_1_ correlated significantly with the ascertained filter lengths (Pearson *r* = −0.8237, *p* = 0.044). Here, the correlation was not significant for PM_10_ and PM_2.5_ (*p* > 0.05). Figure 3 shows the correlations (Pearson) of measured PM mean concentrations with ascertained filter lengths and calculated filling densities of the private-label and brand-name cigarettes.

## 4. Discussion

The PM mean concentrations in SHS from all brands investigated were measured to be very high. This is in line with previous ToPIQ studies [15,16,17,19,20,21] and shows the very high PM burden created by smoking indoors. Children indoors are especially at the mercy of SHS due to their lack of self-determination [23]. This extremely high PM burden caused by smoking underlines the importance of the earliest possible educational advertising regarding the health risks of smoking, e.g., the international Education Against Tobacco (EAT) program [24].

While the three brand-name cigarettes and the reference cigarette showed almost identical PM measurement values, the three private-label brands emitted less PM, with two of those brands emitting only marginally less PM without significance (Table 2). This study does not indicate that cheaper cigarettes emit more PM than more expensive ones. If socially disadvantaged people are assumed to be more likely to smoke private-label cigarettes for financial reasons, this behavior does not expose them to more harmful PM, according to the results presented. However, this needs further checking with additional private-label cigarette brands.

When comparing other characteristics of the six private-label and brand-name cigarettes, it was determined that increased tobacco filling density correlated with reduced PM emission. However, the significance was not on a high level (*p* = 0.01 to 0.05). In a previous study of Indonesian cigarettes, such a correlation was not found, but on the other hand, significant correlations were found both between PM emission and tobacco weight, and between PM emission and nicotine amount [22]. In the present study, a significant correlation with tobacco weight could not be found. Longer filter lengths correlated with lower PM emissions, but only in the case of PM_1_ with relatively low significance (*p* = 0.044). This suggests the effect of cigarette filters on reducing PM emissions. More distinct PM-reducing effects of filters were found in a study about PM emissions from cigarillos [18], whereas another study on PM emissions from cigarettes found that filters had a converse effect [25]. The impact of filters or tobacco-filling densities on PM emissions remains unclear and should be investigated in more detail.

All tested cigarette brands except the reference cigarette had the same tobacco strength (amounts of nicotine, tar, and CO as specified by the manufacturer), so any effects of tobacco strength on PM emissions should be excluded from this study. Reasons for slightly different PM emissions could be the composition of the tobacco or the addition of additives by the manufacturers. These factors could not be clarified by this study due to missing information provided by the manufacturers. A study from 2019 by Braun et al. indicated that additives potentially increase PM concentrations in secondhand smoke [19]. However, Baker et al. in 2004 found no significant influence of additives on PM exposure [26]. In another study, Braun et al. were also unable to prove a correlation between the grade of PM emission and additives [21].

This study, just as in previous ToPIQ studies [16,17,19,21], focused on the comparison of PM emissions from the investigated tobacco products to the emissions of the reference cigarette 3R4F with percentage specifications. The declaration of the absolute measurement data is for comparison to other investigations and guidelines.

According to the WHO, there is no concentration of fine particles that is not harmful [6]. To prevent excessive air pollution from PM, the WHO recommends in their new guidelines from 2021 that the annual mean value of PM_10_ should remain below 15 µg/m^3^ and PM_2.5_ below 5 µg/m^3^. The recommended short-term (24-h) levels are 45 µg/m^3^ for PM_10_ and 15 µg/m^3^ in the case of PM_2.5_ [27]. All tested cigarettes emitted PM in considerable quantities into the environment and exceeded the WHO guidelines many times over. Most particles (>95%) detected were smaller than 1 µm, and can therefore reach deeper regions of the respiratory tract, which can lead to a number of associated health risks [8].

The applied modified smoke protocol differed from the protocol of the International Organization for Standardization (ISO Standard for the Machine Smoking of Cigarettes; 35 mL puff volume, 1 puff/min) [28] and the WHO standard operating procedure for intense smoking of cigarettes (55 mL puff volume, 2 puffs/min) [29], and considered the observation of real smokers [30]. There is no universal standard for smoking regimes [31]. However, the indoor volume (2.88 m^3^) of the applied measuring chamber used in this study is closer to reality than the test volume of smoking machines and is comparable to, e.g., the passenger and cargo volume of a compact car (2.832 to 3.087 m^3^), as stated by US EPA [32]. The data presented here point out the very high PM burden that occurs when smoking in a car with the windows closed and the air conditioner turned off, in particular. Small particles can remain in the air for a very long time, especially without ventilation [33]. The health risks of PM from smoking must be emphasized, and a global ban on smoking in cars is far overdue, partly because car passengers are often children. Children are especially vulnerable, and should be protected from SHS and associated PM exposure, not only in cars, but also in general.

The experimental design with the AETSE differs from real smoking behavior because no real smoker was involved. Due to the severe health damage caused by (passive) smoke, experiments on humans would not have been justifiable. In the respiratory tract of smokers, the inhaled mainstream smoke is humidified. That leads to hygroscopic growth of the exhaled particles 1.5-fold [34]. Additionally, a portion of the inhaled particles remain in the respiratory tract of the smoker [35]. SHS consists of about 85% side-stream smoke and only 15% mainstream smoke [36], and therefore, the AETSE produced tobacco smoke very similar to real SHS without exposing any person. However, the data measured by this study could be slightly too high with a slight bias toward smaller particles if compared to a set-up with real smokers.

The measurement range (0.25 μm to 32 μm) of the LAS Grimm model 1.109 [14] used in this study was a limitation because particles <0.25 µm emitted from the investigated cigarettes could not be detected, although they belong to the PM_1_ fraction as well. Different mean sizes (e.g., 0.1 to 1 µm or 0.02 to 2 µm), and respectively, mean diameters (e.g., 0.1 to 0.5 µm) of tobacco smoke particles were reported [37,38,39,40]. Consequently, most of the particles emitted were detected, and thus, the measurement data are valid. Since the health risks of PM_1_ and ultrafine particles (<0.1 µm) come into focus more [41], a measuring-range extension downwards would be useful.

A higher density of tobacco shops and discounters selling cheaper cigarettes was documented in low-income neighborhoods [42]. Smoking is more widespread in lower socioeconomic groups, which contributes to the social patterning of morbidity [43,44]. A study from Taiwan supports the hypothesis that consumers with lower social status, in particular, are more inclined to smoke private-label cigarettes than to quit smoking if cigarette prices increase [45]. Moreover, youth tend to smoke cheaper cigarettes [46]. High-income smokers were more brand-loyal, whereas smokers with a higher level of education were more likely to switch brands instead of reducing consumption [47]. Since increasing tobacco prices, e.g., via taxes is potentially best intervention to reduce smoking [42], a wider offer of cheaper cigarettes has the opposite effect by facilitating access to tobacco products, even at increasing tobacco prices [48]. During 2002 and 2005, increasing tobacco taxes contributed to an increased share of private-label cigarettes in Canada [49]. It was pointed out that it is important to equalize the sales prices of cigarettes to avoid a market split between cheap and expensive products [50]. Unfortunately, specific taxes that reduce the price gap between cheap and expensive tobacco products have yet to be sufficiently applied [51]. Although, studies revealed the importance of tobacco tax policy, and have highlighted the benefits of specific taxes over ad valorem taxes on tobacco products when aiming to reduce a downward switch to cheaper tobacco products [49,50,52].

## 5. Conclusions

Our investigation of PM amounts in SHS from private-label and more expensive brand-name cigarettes indicates similar, but overall high, PM emissions. This study suggests that cheaper tobacco products are as harmful as more expensive ones, at least regarding PM emissions. Especially for lower socioeconomic groups where smoking is more widespread and cheaper tobacco products are more attractive, anti-smoking campaigns are undoubtedly important. It is also important to inform the public about the PM burden caused by smoking to nonsmokers, which should be the focus of education efforts worldwide.

## Figures and Tables

**Table 1:** Characteristics of the investigated tobacco products. Dimensions and weights are the mean values of five randomized chosen tobacco products of each brand. Amounts of nicotine, tar, and carbon monoxide (CO), as stated by the manufacturers. The number of cigarettes investigated (*n*) after testing for outliers (Grubb’s test) is given in brackets. bn = brand-name cigarette. pl = private-label cigarette. KTRDC = Kentucky Tobacco Research & Development Center. n/a = not applicable.

Brand (*n*)	Reference Cigarette 3R4F (33)	Camel Yellow Filter (34), bn	Marlboro Red (36), bn	Nil Blue (34), bn	Giants Red (38), pl	Goldfield Red (35), pl	Jakordia Red (36), pl
Manufacturer	KTRDC University of Kentucky	JT International GmbH	Philip Morris GmbH Munich	JT International GmbH	Imperial Tobacco Holdings International B.V.	Heintz Van Landewyck GmbH	Johannes Wilhelm von Eicken GmbH
Price per cigarette [EUR]	n/a	0.34	0.34	0.34	0.24	0.24	0.24
Tar [mg]	9.4	10	10	10	10	10	10
Nicotine [mg]	0.73	0.8	0.8	0.8	0.8	0.8	0.8
CO [mg]	12	10	10	10	10	10	10
Total length [mm]	84	84	82	83	83	83	83
Filter length [mm]	27	21	21	22	20	22	25
Filter diameter [mm]	8	8	8	8	8	8	8
Total weight [mg]	980	790	800	800	810	850	860
Tobacco weight [mg]	700	590	590	600	620	640	610
Filling density [mg/cm^3^]	244	186	192	195	196	202	209

**Table 2:** Mean Concentrations (C_mean_) of PM_10_, PM_2.5_, and PM_1_ with standard deviation (SD) of all cigarettes investigated. Percentage variations of C_mean_ of the brand-name (bn) and private-label (pl) cigarettes to C_mean_ of the reference cigarette 3R4F are given in brackets. ns = Not significant (*p* ≥ 0.05). *** = Very significant (*p* < 0.001).

Brand	PM_10_ [µg/m^3^]	PM_2.5_ [µg/m^3^]	PM_1_ [µg/m^3^]
Reference cigarette 3R4F	1655 ± 185	1653 ± 184	1619 ± 178
Camel Yellow Filter (bn)	1635 ± 192 (−1.2%, ns)	1631 ± 191 (−1.3%, ns)	1601 ± 209 (−1.1%, ns)
Marlboro Red (bn)	1686 ± 183 (+1.9%, ns)	1682 ± 182 (+1.8%, ns)	1634 ± 166 (+0.9%, ns)
Nil Blue (bn)	1667 ± 195 (+0.7%, ns)	1662 ± 193 (+0.5%, ns)	1597 ± 174 (−1.4%, ns)
Giants Red (pl)	1591 ± 204 (−3.9%, ns)	1587 ± 203 (−4.0%, ns)	1542 ± 188 (−4.8%, ns)
Goldfield Red (pl)	1577 ± 168 (−4.7%, ns)	1574 ± 167 (−4.8%, ns)	1519 ± 153 (−6.2%, ns)
Jakordia Red (pl)	1394 ± 203 (−15.8%, ***)	1392 ± 202 (−15.8%, ***)	1355 ± 190 (−16.3%, ***)

## Data Availability

Datasets of this study are available from the corresponding author upon request.
